# Dietary *Hermetia illucens* Larvae Replacement Alleviates Diarrhea and Improves Intestinal Barrier Function in Weaned Piglets Challenged With Enterotoxigenic *Escherichia coli* K88

**DOI:** 10.3389/fvets.2021.746224

**Published:** 2021-11-25

**Authors:** Xinxin Jin, Boyu Yuan, Mingming Liu, Mingqiang Zhu, Xue Zhang, Gaijie Xie, Wenxiang Wu, Zifan Wang, Haidong Xu, Yantao Lv, Yanhua Huang, Wei Wang

**Affiliations:** ^1^College of Animal Science & Technology, Innovative Institute of Animal Healthy Breeding, Zhongkai University of Agriculture and Engineering, Guangzhou, China; ^2^College of Veterinary Medicine, Jilin University, Changchun, China; ^3^Department of Pharmacology, College of Basic Medical Science, Jilin University, Changchun, China

**Keywords:** *Hermetia illucens* larvae, weaned piglet, growth performance, intestinal health, immune performance

## Abstract

A high-quality protein substitute, *Hermetia illucens* (black soldier fly) larvae powder, is rich in protein and often used in animal feed. This study aimed to investigate the feasibility and optimal ratio of replacing fish meal with *H. illucens* larvae in weaned piglets and to demonstrate the effects on piglets' growth performance, intestinal microflora and immune performance. Forty-eight female weaned piglets were randomly classified into three groups. Each group consisted of eight pens (replicates), with two piglets per pen. Three groups containing different proportions of *H. illucens* larvae (0, 4, and 8%) were referred to as C, HI4, and HI8. We first designed a 28-day feeding experiment to detect growth performance; after that, the piglets were induced with oral gavage of enterotoxigenic *Escherichia coli* K88 (ETEC K88) and recording diarrhea on day 29 of the experiment. Samples were taken on the 32nd day to detect the effect of *H. illucens* larvae on the immune performance of the weaned piglets. *H. illucens* larvae replacement did not cause any obvious change in the growth performance nether in HI4 nor in HI8 of weaned piglets with 28 d feeding stage. *H. illucens* larvae could improve the intestinal health of weaned piglets by increasing the content of *Lactobacillus* and reducing the content of *Streptococcus*. Compared with C+K88 group, the diarrhea rate was attenuated for the *H. illucens* supplemented group. The integrity of ileum villi in HI4+K88 and HI8+K88 groups was better than that in C+K88 group, and the villi in C+K88 group were severely damaged. The expression of *IL-10, Occludin* and *Claudin-3* in the intestinal mucosa of the HI4+K88 group and HI8+K88 group were significantly increased (*P* < 0.05), and the expression of *TNF*-α was significantly decreased (*P* < 0.05) compared with the C+K88 group. The results of immunoblotting also validated that the same ETEC K88 treatment of weaned piglets enhanced the expression of tight junction protein in the intestinal mucosa of the *H. illucens* addition group. ETEC-induced diarrhea will be reduced by the diet of weaned piglets containing *H. illucens* larvae, ameliorating the immune performance of piglets. Our results indicates that the optimal dosage of *H. illucens* replacement in weaned piglets is 4%.

## Introduction

A saprophytic hydrofidae insect, *Hermetia illucens* (Black soldier fly), can feed on poultry manure and domestic garbage (1). Being rich in protein, amino acids, lauric acid, and minerals, its larvae are a feed material with a high nutritional value. It can be supplemented in livestock and poultry feed production instead of soybean meal (2). Animals exhibited better growth and digestion performances by substituting *H. illucens* larvae meal for soybean meal or fish meal in the feed (3). Triggered by foreign bacteria, *H. illucens* will activate the immune system and produce antimicrobial peptides (AMPs) (4, 5). *H. illucens* larvae contain active antibacterial substances, such as chitin, thereby inhibiting bacterial reproduction and increasing the effect of beneficial bacteria in the intestine (6). A significant impact on the gut microbiota and colon metabolites of finishing pigs was obtained by using *H. illucens* larvae powder as a protein source for pig feed (7). When employed as a source of dietary protein for broiler quails, the saturated fatty acid of quail is increased, and the quail's oxidative emergency state is reduced (8). *H. illucens* larvae are rich in fat, mainly lauric acid (C12:0), which has an inhibitory effect on gram-positive bacteria.

Supplementing *H. illucens* larvae to feed can significantly alter the animal intestinal flora and microbial metabolites (SCFAs), which is beneficial to animal growth (9). Diet modification will also affect the microbiota of the pig's digestive tract. Some bacterial fermentation products such as SCFAs are beneficial to the physiological functions of the intestines, for example, providing energy for epithelial cells, maintaining the morphology and function of colonic epithelial cells, and inhibiting the growth of pathogens (10). Intestinal barrier function genes and protein expression levels are important for intestinal health. ETEC infection affects the expression of aquaporin and ion channel protein (11). Diarrhea in weaned piglets is usually accompanied by intestinal inflammation and barrier damage (12). The changes in cytokine and tight junction protein expression in the intestinal mucosa of weaned piglets are closely related to diarrhea (13). However, information is scarce on whether larvae protects against diarrhea in weaned piglets caused by ETEC.

Similarly, there are limited studies that explore the effect of *H. illucens* larvae powder as a source of feed protein on the growth performance and gut microbes of weaned piglets. Therefore, our experiment employed different proportions of *H. illucens* larvae powder instead of fish meal to investigate its effects on weaned piglets. We first fed the piglets with feed containing *H. illucens* larvae powder, followed by oral ETEC K88. Thereafter, we evaluated the growth performance, immune performance, and intestinal morphology of weaned piglets, explored the effect of ETEC K88 on the expression of aquaporin and ion transporter, and finally, elucidated the positive effects of *H. illucens* feed on the challenge of ETEC K88 to weaned piglets.

## Materials and Methods

This study was approved by the animal ethics committee of Zhongkai University of agricultural and engineering. The protocol was approved by the Medical Experimental Animal Center of Guangdong Province (Permit Number: 12-179).

### Analysis of the Chemical Composition of *H. illucens* Larvae Powder

Five-instar dry *H. illucens* larvae were purchased from Guangzhou AnRuiJie Protection Technology Co., Ltd. (Guangzhou, Guangdong, China). The common nutrients (dry matter, DM; crude protein, CP; ether extract, EE; Ash) in the experimental diet and *H. illucens* larvae were analyzed following the procedures of the Official Association of Analytical Chemists (AOAC) (14). The chitin content was estimated according to Finke (15). The larvae powder was digested with 6 mol/L HCl at 110°C for 24 h and amino acid (AA) concentration was determined by HPLC. According to the AOAC protocols, the concentration of methionine was estimated after oxidation with formic acid, whereas the concentration of tryptophan was obtained after alkaline hydrolysis (14). The chemical composition, energy content, mineral content, and amino acid content are listed in detail in Table 1.

**Table 1:** The main nutrients of *Hermetia illucens* larvae.

**Items**	***Hermetia illucens*** **larvae**
Analyzed composition	
DM, %	94.90
CP, %	33.39
Gross energy, MJ/Kg	22.40
EE, %	40.60
Ash, %	10.90
Chitin, %	3.20
Mineral composition, %	
Total P	0.76
Ca	2.77
Essential amino acids, %	
Lysine	2.00
Methionine+Cystine	0.62
Isoleucine	1.47
Tryptophan	2.35
Valine	1.87
Threonine	1.27
Arginine	1.63
Phenylalanine	1.46
Histidine	1.00
Alanine	2.96
Non-essential amino acids	
Aspartate	2.71
Glutamate	4.80
Glycine	2.20
Serine	1.40
Tyrosine	1.77

### Bacterial Strains

ETEC K88 was original clinically separated and identified (16), and stored in our laboratory. ETEC K88 strains were streaked inoculated on LB plates, and cultured overnight at 37°C. The next day, a single colony was inoculated into LB (Luria-Bertani) broth medium. K88 strains were diluted with PBS (phosphate buffer solution) to ~10^9^ CFU/mL for the challenge by oral gavage at a dose of 50 mL/pig. (17, 18).

### Animal, Diets and Experimental Design

Forty-eight young female weaned piglets (Duroc × Landrace × Large White) with initial body weights (BW) 7.68 ± 0.26 kg were randomly classified into three groups. Each group consisted of eight pens (replicates), with two piglets per pen. The present study investigated three different feeding patterns containing different proportions of *H. illucens* larvae powder (0, 4, 8%) named C, HI4, HI8. The formulas of all experimental diets met or exceeded the nutritional recommendations of the National Research Council 2012 (Table 2). During the feeding period, the piglets were allowed *ad libitum* access to feed and water.

**Table 2:** Composition and nutrient level of the basal diet (as-fed basis).

**Item^a^**	**C group**	**HI4 group**	**HI8 group**
Ingredient, %			
Corn	52.98	51.53	50.08
Soybean meal	9.00	9.00	9.00
*Hermetia illucens* larvae	0	4.00	8.00
Fish meal	4.00	2.00	0
Extruded soybean meal	9.00	9.34	9.68
Soy protein concentrate	6.00	6.00	6.00
Whey powder	10.00	10.00	10.00
Soybean oil	2.00	1.18	0.36
White granulated sugar (sucrose)	2.00	2.00	2.00
DL-Met (99%)	0.36	0.38	0.40
Lys-HCl (78%)	0.80	0.82	0.84
Thr (98%)	0.38	0.38	0.38
Trp (99%)	0.08	0.08	0.08
Stone powder (36%)	0.65	0.44	0.22
Dicalcium phosphate	1.10	1.20	1.30
Choline chloride (50%)	0.20	0.20	0.20
Salt	0.45	0.45	0.45
Premix (multi-dimensional and multi-ore)^b^	1.00	1.00	1.00
Total	100	100	100
Energy and nutrient composition^c^			
DE (MJ/Kg)	14.59	14.59	14.59
CP (%)	19.15	19.15	19.15
Ca (%)	0.82	0.82	0.82
Total P (%)	0.66	0.66	0.66
Lys (%)	1.58	1.58	1.58
Met+Cys (%)	0.90	0.90	0.90
Thr (%)	1.01	1.01	1.01
Trp (%)	0.27	0.27	0.27

a *C is the control diet, diets HI4, HI8 contained 4 and 8% Hermetia illucens larvae in an amount providing similar nitrogen to the diet as control diet, respectively*.

b *Provided per kilogram of complete diet: vitamin A, 12,400 IU; vitamin D 2,800 IU; vitamin E, 130 mg; vitamin K_3_, 5 mg; vitamin B_1_, 3 mg; vitamin B_2_, 10 mg; vitamin B_3_, 40 mg; vitamin B_6_, 8 mg; vitamin B_12_, 0.04 mg; niacin, 45 mg; pantothenic acid, 15 mg; folic acid, 1 mg; biotin, 0.08 mg; Fe (FeSO_4_), 120 mg; Cu (CuSO_4_), 16 mg; I (CaI_2_O_6_), 0.7 mg; Se (Na_2_SeO_3_), 0.48 mg; Zn (ZnSO_4_), 120 mg; Mn (MnSO_4_), 120 mg;*.

c *Calculated values unless indicated otherwise*.

The experiment was carried out for 32 days. For the first 28 days, piglets were fed with different proportions of *H. illucens* larvae powder. The body weight was measured once a week on an empty stomach; the feed intake was recorded every day. Then orally gavage ETEC K88 (50 × 10^9^ CFU/mL) was provided on the 29th day. Eight weaned piglets in each group were randomly selected for the oral gavage experiment.

### Growth Performance

Feed consumption was recorded daily during the trial. At 08:00 on day 1, 8, 15, 22 and 28, all piglets were weighed to determine initial body weight (BW) and final BW. Based on these data, the ADG, ADFI, and F/G were calculated.

### Diarrhea Score

On the second stage after oral administration of ETEC K88 (d 29–32), diarrhea was observed at 08:30 and 16:30 daily; the standard grade score of diarrhea was as follows: solid = 0, semisolid = 1, semiliquid = 2 and liquid = 3 (19). Diarrhea index is the sum of repeated fecal scores during the trial period / total number of repeated feeding during the trial period. The number of piglets with diarrhea was determined and used to calculate the incidence of diarrhea, i.e., the ratio of the number of piglets with diarrhea to the total number of piglets.

### Sampling and Processing

At 08:00 on the 25th day of the trial, the feces of each group were also collected for 16S rRNA detection. Polyvinyl chloride plastic bags were attached to the anus of weaned piglets to prevent fecal contamination and falling off on the ground. Feces were put in centrifuged tubes quickly and saved in liquid nitrogen. On day 28 of the trial, and 5 mL of blood was collected from the anterior vena cava, and serum was separated for serum biochemical detection.

After fasting for 12 h before slaughter, on the 32nd day of the experiment, 5 mL of blood was collected. Antioxidant enzymes and immunoglobulin in serum were detected by ELISA. Four pigs in each group were euthanized and slaughtered. The intestinal tissue was isolated and washed with PBS, the intestinal tissue was collected, and the intestinal mucosa was scraped (20). A part of the ileum tissue was fixed in 4% formaldehyde for subsequent section analysis. Other samples were then quickly frozen in liquid nitrogen and stored in an ultra-low temperature refrigerator at −80°C for future use.

### Serum Parameters

Eight serum samples were collected from each group on day 28. The serum biochemical indicators including TP, ALB, GLB, AST, ALT, TG, TC, UREA, P, and Ca. were evaluated by automatic biochemical analyzer BS-240VET (Mindray Bio, Shenzhen, China).

### Analysis of Ileum Morphology

The fixed ileum segment was dehydrated, paraffin embedding was performed, sectioned for intestinal morphology, and the sections were submitted to Hematoxylin & Eosin staining (21). Images were obtained using a Leica microscope (DM500, Leica, Wetzlar, Germany). Image-Pro Plus 6.0 software (Media Cybernetics, Rockville, MD) was used to measure the villus height (Vh) and crypt depth (Cd). Morphometric measurement of 10 well-oriented and intact villi and 10 crypts selected from the ileum (22).

### DNA Extraction and Sequencing

Feces samples from each group of eight piglets were collected for nucleic acid extraction. Total DNA in the samples was extracted using the RNeasy Power Microbiome KIT (Qiagen, Milan, Italy), following the manufacturer's instructions. The 16S rRNA gene V3–V4 region was amplified by gDNA to evaluate the microbiota (23). PCR products were tested on the Illumina MiSeq platform (Majorbio, Shanghai, China), following the instructions (24).

### Antioxidant Enzyme Activity Determination and Immunoglobulin Determination

Antioxidant enzyme activity in the serum was measured using a commercial assay kit (Nanjing Jiancheng Biological Engineering Institute, Nanjing, China), such as CAT (Catalase, A007–1–1), POD (Peroxidase, A084–2–1), strictly following the manufacturer's instructions. The manufacturer's instructions were strictly followed while using Porcine IgG ELISA KIT and Porcine IgA ELISA KIT (LJ-871426, LJ-871428 Lingjiang Biotechnology, Guangzhou, China).

### RNA Extraction From Intestinal Mucosa and Quantitative PCR

Of jejunal mucosa, ileum mucosa and colon mucosa, 0.1 g of the sample was taken, and autoclave magnetic beads were added. Tissues were homogenized with a Tissue Homogenizer and total RNA extracted by TRIzol reagent method (Takara Biotechnology, Dalian, China) (25). The quality of RNA was estimated with a microspectrophotometer Q3000 (Quawell Technology, Inc., USA) and Ratio (OD260: OD280) from 1.8 to 2.0. RNA (1 μg) was reverse transcribed into cDNA with the Synthesis Kit (Takara Biotechnology, Dalian, China). Real-time PCR of the target genes and GAPDH was conducted on CFX96 Real-Time PCR Detection System (Bio-Rad, Hercules, CA, USA) with TB Green™ Premix Ex Taq™ (Takara Biotechnology, Dalian, China). The primers related to the evaluation of the immune status and the tight junctions are detailed in Table 3. Reagents were added according to the operating instructions. The circulation parameters used were as follows: 95°C for 30 s; 40 cycles of 95°C for 5 s, 60°C for 34 s; 95°C for 15 s; 65°C for 5 s; 95°C for 5 s. The expression levels of each target gene were normalized based on the housekeeping gene GAPDH, according to the following formula 2^−(ΔΔCt)^, where ΔΔCt = (Ct_target_ – Ct_GAPDH_)_treatment_ – (Ct_target_ – Ct_GAPDH_)_control_.

**Table 3:** Primers for real-time PCR.

**Gene**	**Sequences**	**Length (bp)**
*IL-8*	Forward: 5′-CCAGTGCATAAATACGCATTCCA −3′	138
	Reverse: 5′-GGGTCCAGGCAGACCTCTTTT −3′	
*IL-10*	Forward: 5′-GACCAGATGGGCGACTTGTTG −3′	160
	Reverse: 5′-TCGGCTTTGACATTGGCTAC −3′	
*TNF-α*	Forward: 5′-GCCTCTTCTCCTTCCTCCTG −3′	193
	Reverse: 5′-TCGGCTTTGACATTGGCTAC −3′	
*IFN-γ*	Forward: 5′-TCCAGCGCAAAGCCATCAGTG−3′	111
	Reverse: 5′-ATGCTCTCTGGCCTTGGAACATAGT−3′	
*ZO-1*	Forward: 5′-GCCATCCACTCCTGCCTAT−3′	133
	Reverse: 5′-CGGGACCTGCTCATAACTTC−3′	
*Occludin*	Forward: 5′-TGGCTGCCTTCTGCTTCATTGC−3′	131
	Reverse: 5′-GAACACCATCACACCCAGGATAG−3′	
*Claudin-3*	Forward: 5′-GCCAAAGCCAAGATCCTCTA−3′	87
	Reverse: 5′-GTAGTCCTTGCGGTCGTAGG−3′	
*AQP1*	Forward: 5′-GACACCTGCTGGCGATTGACTAC−3′	90
	Reverse: 5′-GGTCCTGGAAGTTGTGCGTGATC−3′	
*AQP3*	Forward: 5′-TGACCTTCGCTATGTGCTTCC−3′	212
	Reverse: 5′-GTCCAAGTGTCCAGAGGGGTAG−3′	
*AQP7*	Forward: 5′-CCCGTGCCTCCAAGATGA-3′	58
	Reverse: 5′-CGCATTATTGTTTGCATCTTTGA-3′	
*AQP9*	Forward: 5′-TGTCATTGGCCTCCTGATTG-3′	62
	Reverse: 5′-TGGCACAGCCACTGTTCATC-3′	
*NKCC1*	Forward: 5′-CCAATGCTGTTGCAGTTGCT-3′	364
	Reverse: 5′- TGGGCTTCTTGCTCTCCAAG-3′	
*NHE3*	Forward: 5′-AGCTGGAGATCATAGACCAGGT−3′	147
	Reverse: 5′-CGGTGAAGAAGATGACGATGAG−3′	
*CFTR*	Forward: 5′-ACTATGGACCCTTCGAGCCT−3′	123
	Reverse: 5′-CGCATTTGGAACCAGCGTAG−3′	
*GAPDH*	Forward: 5′-GCCATCACTGCCACCCAGAA−3′	153
	Reverse: 5′-GCCAGTGAGCTTCCCGTTGA−3′	

*IL, Interleukin; TNF-α, Tumor necrosis factor-α; IFN-γ, Interferon-γ; ZO-1, Zona occludens protein 1; AQP, aquaporins; NKCC1, Na-K-Cl cotransporter; NHE3, Na+/H+ exchanger 3; CFTR, cystic fibrosis transmembrane conductance regulator*.

### Western Blotting

The jejunal mucosa, ileum mucosa, and colon mucosa of pigs were homogenized in tissue lysate containing protease inhibitors. The homogenate was incubated at 4°C for 30 min to promote lysis. The lysate was centrifuged at a speed of 12,000 r/min for 20 min, and the supernatant collected served as the total protein for the western blotting. Subsequently, Western blotting was performed according to the standard protocol (26). β*-Actin* antibody (1:1,000; CST, 13E5–4970), *Occludin* (1:1,000; Abcam, ab167161), *Claudin-3* (1:1,000; Abcam, ab15102), goat anti-rabbit IgG (1:5,000, Boster, BA1055) were used.

### Statistical Analysis

The data of growth performance, serum biochemical index were analyzed using the IBM SPSS Statistics V25.0 software (SPSS Inc., Chicago, IL, USA). One-way analysis of variance (ANOVA) was used for linear and quadratic curve analysis, and Duncan's method was used for multiple comparisons to evaluate growth performance and serum biochemical indicators. The results of qPCR and western blotting were reflected as mean ± standard deviation (SD). Quantitative PCR and western blotting were triplicates, and the representative results were shown. Statistical analysis was done with the help of one-way analysis of variance (ANOVA), analyzed using GraphPad Prism 7.0 (GraphPad Software, San Diego, CA). The *P*-value < 0.05 was considered statistically significant.

## Results

### Effect of Dietary *H. illucens* Larvae on Growth Performance of Weaned Piglets

The animals were in good health since the initiation of the experiment. In this experiment, the piglets were growing well without death. The ADG, ADFI, and F/G of each group of piglets are given in Table 4. There was no difference among the three groups in ADG, ADFI, F/G for 1–28 days. However, in 8–14 days, the F/G of the HI4 group was significantly lower than that of other groups (*P* < 0.05); a significant difference was also noted, in 22–28 days, between the F/G of the HI4 and HI8 groups from the C group (*P* < 0.05), which showed a linear response to increasing H. illucens larvae levels. All these results indicates that replace fish meal with H. illucens larva meal either with 4 or 8% in weaned piglets diet sustain a similar growth performance.

**Table 4:** The ADG, ADFI, and F/G conditions of weaned piglets fed with different proportions of *Hermetia illucens* larvae for 7, 14, 21, 28 d (*n* = 16).

**Items**	**C group**	**HI4 group**	**HI8 group**	***P*** **-value**		
				**Inter-group**	**Linear**	**Quadratic**
**1–7 d**						
ADG/g	209.05 ± 66.8	180.00 ± 60.80	181.91 ± 56.55	0.360	0.234	0.431
ADFI/g	319.81 ± 64.23	277.91 ± 55.81	304.67 ± 55.33	0.417	0.635	0.222
F/G	1.53 ± 0.30	1.61 ± 0.32	1.67 ± 0.30	0.691	0.396	0.972
**8–14d**						
ADG/g	268.57 ± 83.37	282.86 ± 102.61	240.48 ± 59.86	0.379	0.364	0.291
ADFI/g	480.57 ± 38.29^a^	452.48 ± 32.79^ab^	436.67 ± 28.45^b^	0.070	0.024	0.696
F/G	1.79 ± 0.14^a^	1.59 ± 0.11^b^	1.82 ± 0.11^a^	0.009	0.669	0.003
**15–21 d**						
ADG/g	337.10 ± 94.46	283.33 ± 96.70	267.14 ± 91.54	0.116	0.048	0.531
ADFI/g	551.14 ± 48.78^a^	512.76 ± 41.76^ab^	494.48 ± 26.02^b^	0.047	0.017	0.589
F/G	1.65 ± 0.14^b^	1.80 ± 0.14^a^	1.85 ± 0.09^a^	0.022	0.009	0.336
**22–28d**						
ADG/g	287.14 ± 133.66	292.86 ± 79.08	345.20 ± 111.28	0.715	0.444	0.779
ADFI/g	644.00 ± 39.32	597.05 ± 39.45	602.03 ± 40.88	0.079	0.064	0.177
F/G	2.24 ± 0.13^a^	2.03 ± 0.13^b^	1.76 ± 0.13^c^	0.001	0.0001	0.514
**1–28 d**						
Initial BW/kg	7.68 ± 0.78	7.67 ± 0.83	7.69 ± 0.75	0.999	0.991	0.963
Final BW/kg	15.38 ± 2.89	14.90 ± 2.33	14.90 ± 2.22	0.832	0.604	0.758
ADG/g	274.88 ± 51.99	257.98 ± 56.93	250.88 ± 55.12	0.820	0.550	0.887
ADFI/g	498.88 ± 136.86	460.05 ± 135.13	459.52 ± 123.89	0.891	0.683	0.818
F/G	1.80 ± 0.31	1.76 ± 0.21	1.77 ± 0.08	0.970	0.873	0.756

*In the same row, values with no letter or the same letter superscripts mean no significant difference (P > 0.05), while with different letter superscripts mean significant difference (P < 0.05). Each group consisted of eight pens (replicates), with two piglets per pen*.

### Effect of Dietary *H. illucens* Larvae on Serum Biochemical Parameters of Weaned Piglets

Table 5 demonstrated non-significant differences in GLB and AST between the three groups; however, the TP content of the HI4 group and HI8 group was significantly different from the C group (*P* < 0.05). *H. illucens* larvae powder significantly increased the content of phosphorus and calcium in serum (*P* < 0.05) compared with the C group. TP, ALB, ALT, TG, TC, UREA, and Ca, which showed a linear response, respectively, to increasing *H. illucens* meal levels (*P* < 0.05, with the maximum corresponding to the inclusion of 8% of *H. illucens* meal). *P* which showed a linear and quadratic response to increasing *H. illucens* meal levels (*P* < 0.05, with the maximum corresponding to the inclusion of 4% *H. illucens* meal).

**Table 5:** Effects of *Hermetia illucens* larvae on serum biochemical parameters of weaned piglets (*n* = 8).

**Items**	**C group**	**HI4 group**	**HI8 group**	***P*** **-value**		
				**Inter-group**	**Linear**	**Quadratic**
TP (g/L)	45.96 ± 3.91^b^	53.50 ± 3.69^a^	54.56 ± 7.18^a^	0.006	0.003	0.164
ALB (g/L)	26.07 ± 3.54^b^	29.69 ± 3.52^ab^	30.76 ± 3.91^a^	0.046	0.018	0.133
GLB (g/L)	19.88 ± 3.58	23.81 ± 3.85	23.80 ± 5.81	0.161	0.099	0.327
AST (U/L)	44.66 ± 3.67	44.63 ± 3.59	45.05 ± 3.48	0.424	0.225	0.642
ALT (U/L)	44.76 ± 9.25^b^	51.71 ± 8.83^ab^	69.10 ± 26.44^a^	0.026	0.009	0.485
TG (mmol/L)	0.25 ± 0.06^b^	0.27 ± 0.07^b^	0.37 ± 0.11^a^	0.026	0.014	0.226
TC (mmol/L)	1.70 ± 0.26^b^	1.96 ± 0.14^ab^	2.18 ± 0.41^a^	0.013	0.003	0.903
UREA (mmol/L)	2.53 ± 0.61^b^	2.74 ± 0.86^ab^	3.42 ± 0.87^a^	0.086	0.036	0.490
P (mmol/L)	2.36 ± 0.44^b^	3.07 ± 0.26^a^	2.90 ± 0.39^a^	0.003	0.009	0.013
Ca (mmol/L)	2.00 ± 0.23^b^	2.32 ± 0.15^a^	2.36 ± 0.22^a^	0.004	0.002	0.129

*In the same row, values with no letter or the same letter superscripts mean no significant difference (P > 0.05), while with different letter superscripts mean significant difference (P < 0.05). TP (Crude protein), ALB (Albumin), GLB (Globulin), AST (Aspartate aminotransferase), ALT (alanine aminotransferase), TG (Triglyceride), TC (Total cholesterol), P (Phosphorus), Ca (Calcium), and UREA*.

### Effects of Dietary *H. illucens* Larvae on the Composition and Diversity of Fecal Microorganisms in Weaned Piglets

**Figure 1:**
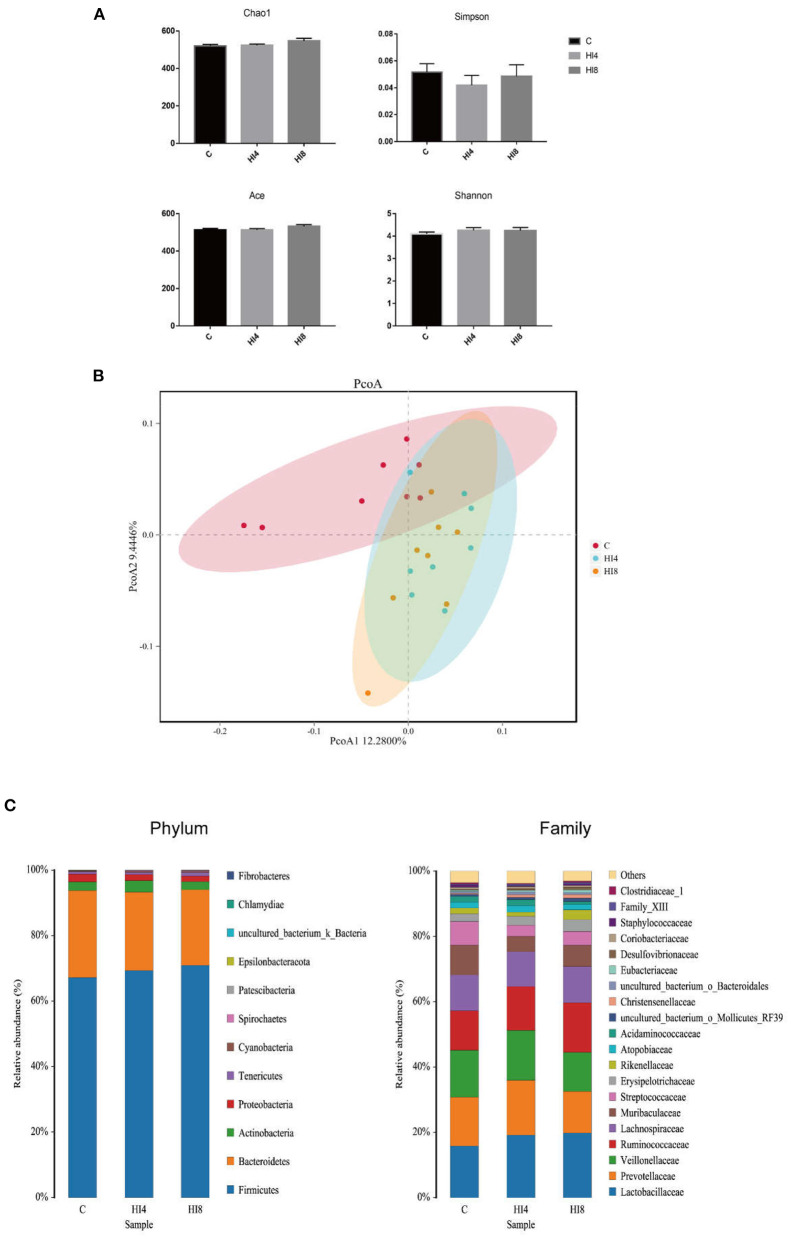
Richness and diversity of fecal. **(A)** Chao1 index, Simpson index, Ace, Shannon. The values are means ± SEM (*n* = 8). **(B)** Principal coordinates analysis (PcoA) of bacterial communities in the fecal of pigs (based on the Binary-Jaccard). (*n* = 8). **(C)** Phylum-level and Family-level relative abundance of 16S rRNA gene sequences from the fecal of pigs (*n* = 8). The figure provided is an average of the three analyses.

16S rRNA Illumina MiSeq sequencing revealed the microbial composition in the feces of the piglets treated by *H. illucens* larvae. In this study, 65863 effective sequences from 24 samples were screened for subsequent analysis, with an average of 482 operation taxonomic units (OTUs) per sample in fecal. As illustrated in Figure 1A, there was no significant difference in Shannon, Ace, Chao1, Simpson. However, the PCoA with the binary-Jaccard distance results validated that the HI4 group and HI8 group were separate from the C group (Figure 1B). The outcome of the analysis between the groups using the PERMANOVA test revealed that *R*^2^ = 0.17, *P* = 0.001.

At the phylum level (Figure 1C), Firmicutes and Bacteroidetes are two advantageous categories, the contents of Firmicutes in the C group, HI4 group, and HI8 group were 67.21, 69.34, and 70.92%, respectively. The contents of Bacteroidetes in the C group, HI4 group, and HI8 group were 26.55, 23.94, and 23.07%, respectively. The next two most dominant phyla, Proteobacteria and Actinobacteria, accounted for 2.41 and 2.63% in the C group, 1.84 and 3.52% in the HI4 group, and 1.68 and 2.48% in the HI8 group, respectively.

**Figure 2:**
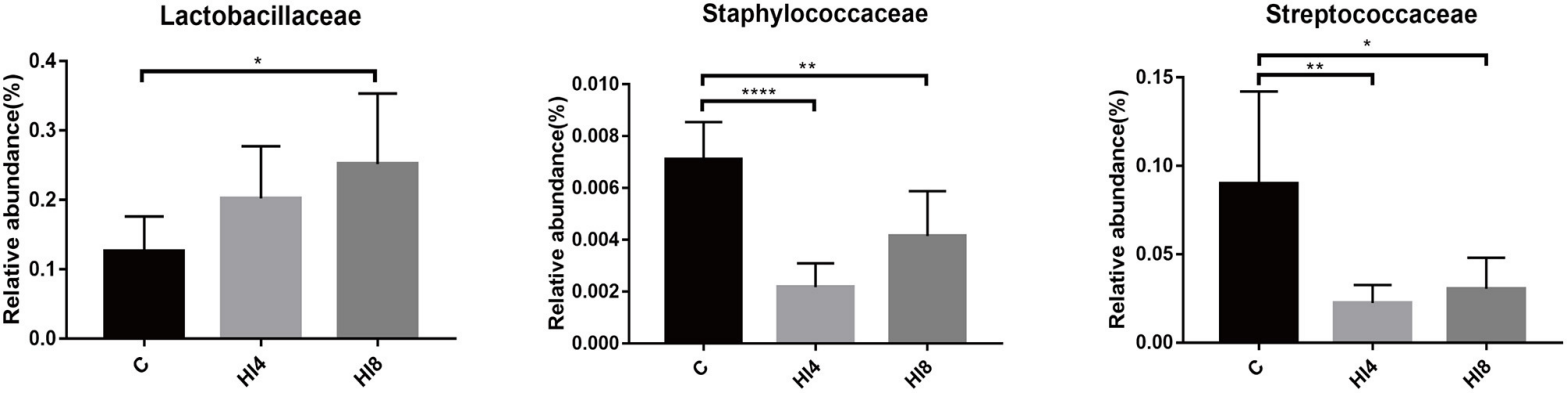
Significantly changed bacterial family. The values were expressed as the mean ± SD (One-way ANOVA with a Tukey *post-hoc* test): *P < 0.05, **P < 0.01, ***P < 0.001, ****P < 0.0001, (*n* = 8). The figure provided is an average of three analyses.

At the family level, the content of Lactobacillaceae in the HI4 group and HI8 group was higher than that in the C group; the most significant difference was reflected between HI8 and C groups (*P* < 0.05). On the other hand, significantly lower content of both Streptococcaceae (*P* < 0.05) and Staphylococcaceae (*P* < 0.01) was evident in the HI4 group and HI8 group than that in the C group (Figure 2).

The 10 most predominant genera at the genus level were *Lactobacillus, Megasphaera, Prevotella*, uncultured_Muribaculaceae, *Streptococcus, Agathobacter, Subdoligranulum, Prevotella_7*, uncultured_Prevotellaceae, uncultured_Veillonellaceae. The levels of *Lactobacillus* in the HI4 group and HI8 group were higher than those in the C group. In contrast, the levels of *Streptococcus* were significantly lower than those in the C group (*P* < 0.05). The relative abundance of 16S rRNA gene sequence in pig feces was represented in Supplementary Figure 1, and the heat map portrayed the 20 species of bacteria most dominant at the genus level in the feces.

### Effect of Dietary *H. illucens* Larvae on Diarrhea Rate of Weaned Piglets Challenged With ETEC K88

After inoculating piglets with ETEC K88, each group manifested different rates of diarrhea. As depicted in Table 6, the incidence of diarrhea was 50% in the C+K88 group, 37.5% in the HI4+K88 group, and 25% in the HI8+K88 group.

**Table 6:** Effect of *Hermetia illucens* larvae on diarrhea rate and diarrhea index of weaned piglets after intragastric administration of ETEC K88.

**Item**	**C+K88**	**HI4+K88**	**HI8+K88**
Incidence of diarrhea (%)	50%	37.5%	25%
Diarrhea Index	1.67	1.25	0.83

### Effect of Dietary *H. illucens* Larvae on Ileum Morphology of Weaned Piglets Challenged With ETEC K88

**Figure 3:**
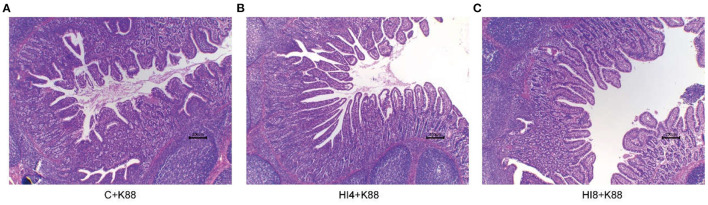
Representative ileum morphology of different group piglets. **(A)** Morphology of ileum villi in C+K88 group. **(B)** Morphology of ileum villi in C+K88 group. **(C)** Morphology of ileum villi in C+K88 group. Representative optical micrographs of intestinal cross-sections. Scale bar = 200 μm. *n* = 3.

The morphology of the ileum is sketched in Figure 3 and Table 7. We made paraffin sections of the ileum and found that the intestinal morphological integrity of the HI4+K88 and HI8+K88 groups was superior to that of the C+K88 group. As observed in Figure 3, the ileum villi in the C+K88 group witnessed the most severe damage with shortened and blunt, deep crypts. However, the integrity of ileum villi in the HI4+K88 and HI8+K88 groups was significantly different from that in the C+K88 group. As described in Table 7, comparing the Vh (villus height) and Cd (Crypt depth) of the ileum in the C+K88 group, the Vh was found to be significantly increased in HI4+K88 and HI8+K88 groups (*P* < 0.05), whereas there was no significant difference in the Cd. Moreover, the villus height to crypt depth ratios in the ileum in the HI4+K88 and HI8+K88 groups were significantly higher than that in the C+K88 group (*P* < 0.05).

**Table 7:** Effect of *Hermetia illucens* larvae on ileum morphology of piglets attacked by ETEC K88 (*n* = 4).

**Item**	**C+K88**	**HI4+K88**	**HI8+K88**	***P*** **-value**
Vh, μm	258.75 ± 38.50^b^	451.79 ± 80.79^a^	427.79 ± 129.06^a^	0.001
Cd, μm	405.38 ± 56.47	381.87 ± 83.74	428.68 ± 76.11	0.372
Vh/Cd, μm	0.65 ± 0.14^b^	1.22 ± 0.31^a^	1.03 ± 0.37^a^	0.001

*In the same row, values with no letter or the same letter superscripts mean no significant difference (P > 0.05), while with different letter superscripts mean significant difference (P < 0.05). The data presented are the average of three independent experiments*.

### Effect of Dietary *H. illucens* Larvae on the Expression of Ion Transporter and Aquaporin in the Ileum Mucosa of Weaned Piglets Challenged With ETEC K88

**Figure 4:**
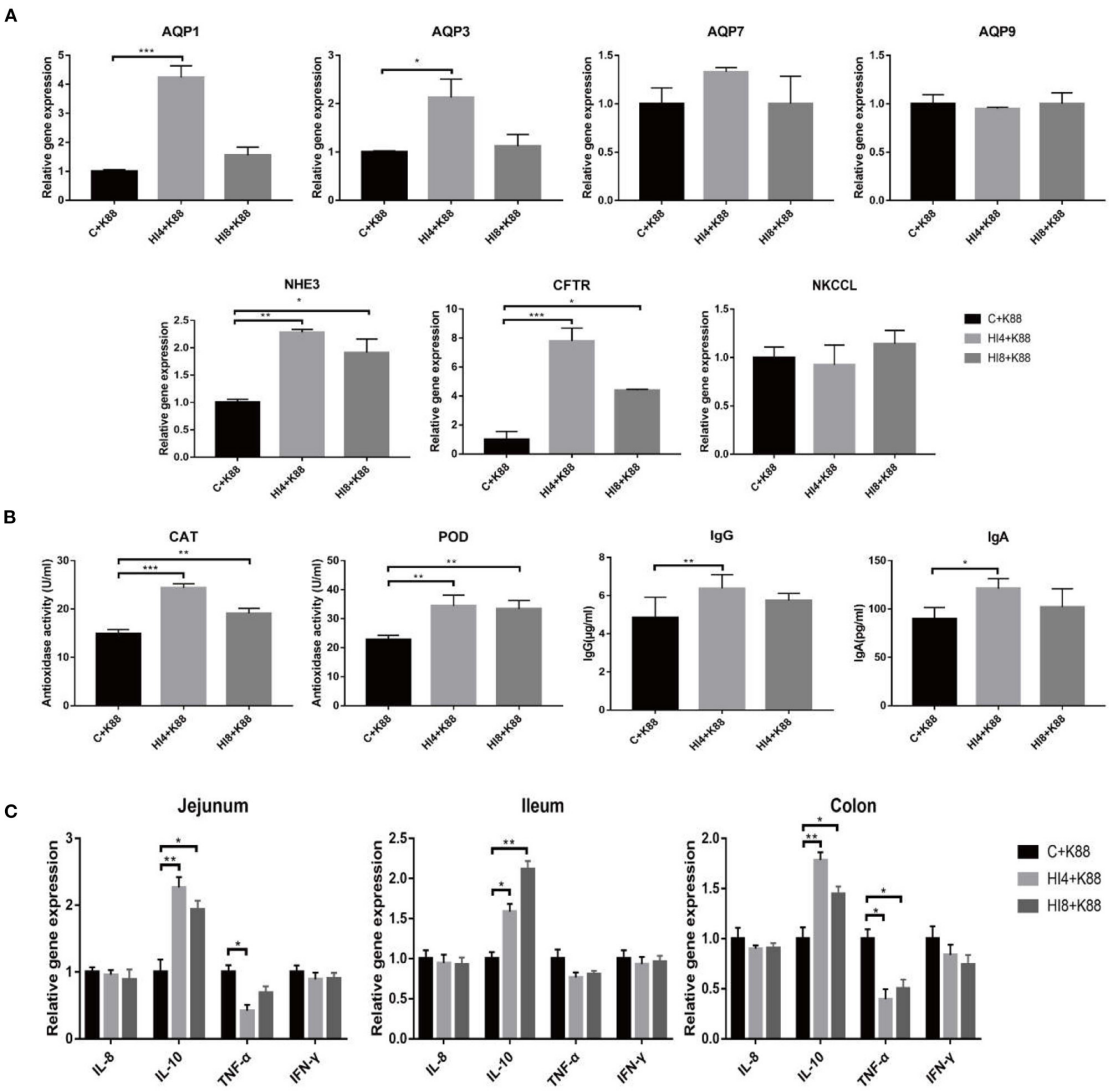
The challenge of ETEC affects the expression of ion transporter and aquaporin in the ileal mucosa of piglets, the content of antioxidant enzymes and immunoglobulins in serum, and the expression of inflammatory factors in the intestinal mucosa. **(A)** The relative mRNA expression of intestinal ion transporters and aquaporins (*AQP*) in ileum mucosa. *AQP*, aquaporins; *CFTR*, cystic fibrosis transmembrane conductance regulator; *NHE3*, Na+/H+ exchanger 3; *NKCC1*, Na-K-Cl cotransporter. Results are mean ± SD (*n* = 4). **(B)** Antioxidant enzyme activity and immunoglobulin concentration in serum of challenged weaned piglets (One-way ANOVA with a Tukey *post-hoc* test) (*n* = 4). **(C)** Effects of dietary supplementation of *Hermetia illucens* larvae on real-time mRNA expression of cytokines and barrier function genes in jejunal mucosa, ileum mucosa, colon mucosa (One-way ANOVA with a Tukey *post-hoc* test) (*n* = 4). *P < 0.05, **P < 0.01, ***P < 0.001, (*n* = 4). The presented data are the average of three independent experiments.

As substantiated in Figure 4A, the mRNA expression of *NHE3* and *CFTR* was increased significantly in the ileum mucosa of the ETEC K88 challenged piglets belonging to the HI4+K88 and HI8+K88 groups (*P* < 0.05) as compared to the C+K88 group. Among these, the difference in the HI4+K88 group was the most significant.

The results of AQP1 and AQP3 are demonstrated in Figure 4A. Compared with the C+K88 group, the expression of *AQP1* and *AQP3* in the HI4+K88 group was significantly different (*P* < 0.05); however, the HI8+K88 group was insignificant. Nevertheless, there is no difference between the three groups of *AQP7, AQP9*, and *NKCCL*.

### Effect of Dietary *H. illucens* Larvae Powder on the Antioxidant Enzyme Activity and Immunoglobulin Content of Weaned Piglets Challenged With ETEC K88

We estimated the activities of peroxidase and catalase in serum on day 32 of the experiment. As shown in Figure 4B, the CAT activity of the HI4+K88 and HI8+K88 groups significantly differed from the C+K88 group (*P* < 0.05). The result of POD activity was parallel to that of CAT; both the *H. illucens* larvae addition group was significantly higher than the control group (*P* < 0.05). Compared with the C group, the IgG and IgA concentrations of the HI4+K88 group exhibited significant differences (*P* < 0.05). Though there was no significant difference in the HI8+K88 group, there was a distinguished tendency to increase.

### Effect of Dietary *H. illucens* Larvae on Gene Expression of Inflammatory Factors in the Intestinal Mucosa of Weaned Piglets Challenged With ETEC K88

To evaluate the impact of feeding *H. illucens* larvae on intestinal immune function, we elucidated the mRNA expression of several inflammatory factors.

As shown in Figure 4C, compared with C+K88 group, the expression of *IL-10* in the jejunum, ileum, and colon mucosa of HI4 +K88 group and HI8 +K88 group was significantly increased (*P* < 0.05). Compared with C+K88 group, the expression of *TNF-*α in the jejunum mucosa was significantly decreased in HI4 +K88 group (*P* < 0.05). The expression of *TNF-*α in colon mucosa of HI4+K88 group and HI8+K88 group was significantly decreased compared with that of C+K88 group (*P* < 0.05). However, there were no significant differences in the expression of *IL-8* and *IFN-*γ in the jejunum, ileum, and colon mucosa of the three groups of piglets.

### Effect of Dietary *H. illucens* Larvae on the Expression of Tight Junction Proteins in the Intestinal Mucosa of Weaned Piglets Challenged With ETEC K88

**Figure 5:**
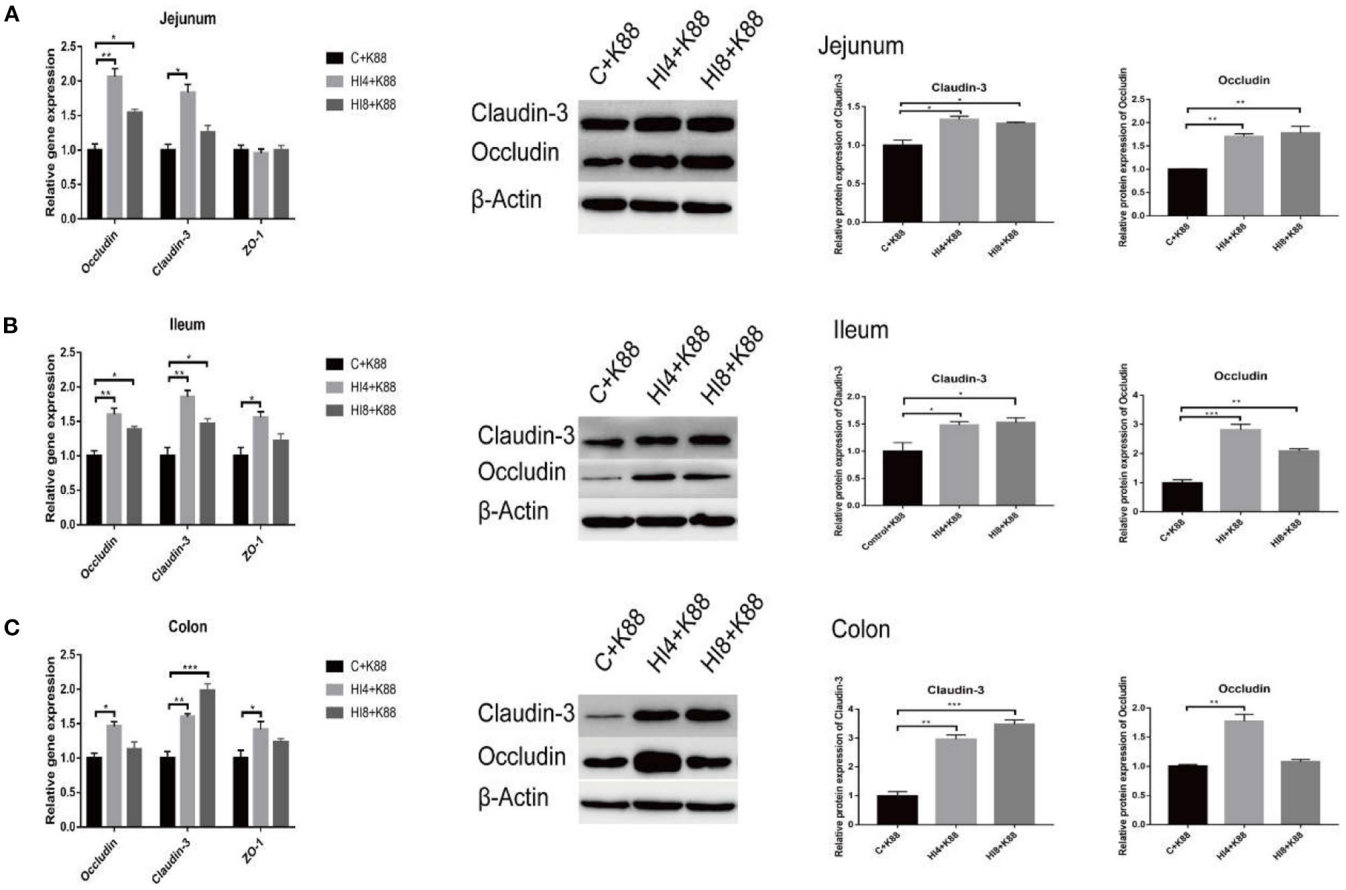
Effects of dietary supplementation of *Hermetia illucens* larvae on real-time mRNA expression and protein of barrier function genes in jejunal mucosa, ileum mucosa, colon mucosa. **(A)** Representative immunoblots and quantification of *Claudin-3*, and *Occludin* expression in the jejunum (*n* = 3). **(B)** Representative immunoblots and quantification of *Claudin-3*, and *Occludin* expression in the ileum. **(C)** Representative immunoblots and quantification of *Claudin-3*, and *Occludin* expression in the colon. *P < 0.05, **P < 0.01, ***P < 0.001, (*n* = 3). Data are based on an average of three independent experiments.

As evident in Figure 5A, a significant increase in the mRNA expression of *Occludin* and *Claudin-3* in the jejunum mucosa (*P* < 0.05) of the HI4+K88 group compared with the C+K88 group was observed. However, in the HI8+K88 group, the mRNA expression level of *Occludin* was significantly increased than that in the C+K88 group (*P* < 0.05). The expression levels of tight junction proteins *Occludin, ZO-1*, and *Claudin-3* in the ileum mucosa of the HI4+K88 group manifested a significant increase than those in the C+K88 group (*P* < 0.05) (Figure 5B). The *Occludin* and *Claudin-3* expressions were significantly increased in the HI8+K88 group when compared with that in the C+K88 group (*P* < 0.05) (Figure 5B). Results from the colon mucosa revealed a significant increase (*P* < 0.05) in the tight junction proteins *Occludin, Claudin-3*, and *ZO-1* in the HI4+K88 group, whereas, a significant increase (*P* < 0.05) in only *Occludin* in the HI8+K88 group, in comparison to the C+K88 group (Figure 5C).

The results of western blotting substantiated the results of fluorescence quantification. *Claudin-3* and *Occludin* were significantly increased in the jejunum mucosa, ileum mucosa, and colon mucosa in the HI4+K88 group and HI8+K88 group compared with the C+K88 group (*P* < 0.05).

## Discussion

With a worldwide shortage of resources, *H. illucens* larvae can evolve as a novel feed protein source, widely applied in animal diets. This can make an important contribution to the sustainable development of the livestock industry, facilitating the protection of the ecological environment (27–29).

This study provides certain guiding significance for production and application. It shows promise to completely replace fish meal with *H. illucens* larva meal in weaned piglets diet after weighing a possible positive effect on gut-barrier functionality as it sustain a similar growth performance. However, there is a tendency to decrease F/G in a later stage. The digestibility and absorption rate of P and Ca of weaned piglets in *H. illucens* larval powder supplemental group were enhanced, which may be related to the presence of calcium and phosphorus in the larval powder of *H. illucens* (30). The results of this experiment showed that *H. illucens* larvae powder could enrich the protein absorption capacity of piglets, but this is contrary to the results of Biasato (30). The result agrees with Spranghers's study and provides a reference for the future application of insect protein (31).

The presence of antibacterial peptides and chitosan in *H. illucens* larvae powder ameliorates the resistance of weaned piglets to disease. Marino's research has shown that *H. illucens* larvae powder improves the antioxidant capacity of fish (32). On encountering pathogenic microorganisms, the piglet's immune system will be activated and produce corresponding antibodies. Thus, a higher concentration of IgG and IgA reflects the improved performance of the piglet's immune system. The intestinal morphology findings signify that improved resistance to ETEC K88 and protection to intestinal health may be attributed to the early feeding of *H. illucens* larvae powder.

Enterotoxigenic *Escherichia coli* is the predominant cause of diarrhea in weaned piglets. Oral gavage of ETEC K88 was used to verify the immunity and disease resistance of piglets and the ability to resist the inflammatory response of ETEC K88 infection. The increase in enterotoxigenic *E. coli* affects intestinal permeability, alters the diversity and composition of the microbiota, and induces inflammation by regulating the expression of inflammatory factors, resulting in intestinal inflammation (33, 34). The pathophysiology of ETEC-mediated diarrhea recognized reduced absorptive surface epithelial cells, destruction of tight junction barrier function, impairment of ion transport, coupled with induction of inflammation (35). Severe damage in the ileum villi of the C+K88 group indicated that ETEC K88 is detrimental to the villus structure of the small intestine (36, 37). Vh and Vh/Cd were usually used as criteria for evaluating intestinal mucosal barrier function and intestinal health (38).

The Vh and the Vh/Cd of ileum in the HI4+K88 and HI8+K88 groups were significantly higher than that in the C+K88 group. This may be attributed to the beneficial biomass, such as chitosan and lauric acid, resisting the invasion of ETEC K88. (39, 40). *Occludin* and *Claudin-3* play an important regulatory role in the intestinal barrier (41). Henceforth, up-regulation or down-regulation of these tight junction proteins in the body is closely associated with the intestinal barrier (42, 43). Our results also indicated that the *H. illucens* larval feeding group might have a counteracting effect on ETEC K88's inhibition of *AQP* and ion transporter expression (11, 44). This study indicates that the early feeding of *H. illucens* larvae powder feed may protect intestinal health by stimulating the expression of tight junction proteins in the mucosa when invaded by pathogenic bacteria. This study also demonstrated a positive correlation between the *Claudin-3* and *Occludin* expression with the intestinal epithelial barrier function.

As feeding nutrition is one of the critical key factors that shape the gut microbial ecology, it increases *Lactobacillus*. In contrast, reduced *Streptococcus* was observed in piglets fed with *H. illucens* feed for 4 weeks in this experiment. This was in accordance with the results of Miaoyu's research in finishing pigs (7). Such dietary-induced shifts in gut microbial ecology may affect interactions among major phyla and lead to potential implications in piglets physiology. *Lactobacillus* imparts a protective effect on the intestinal epithelial barrier damage of IPEC-1 cells caused by ETEC and escalates the expression of tight junction proteins *ZO-1* and *Occludin* (45). As expected, compared with the control group, the diarrhea rate in the HI4 and HI8 groups was reduced in this experiment, the intestinal morphology integrity was better, the activity or concentration of CAT, POD, IgG, IgA in the serum was elevated, the expression of anti-inflammatory factors, tight junction proteins increased. The pro-inflammatory factors decreased, intestinal health and immune performance were ameliorated, and barrier function was better maintained.

*H. illucens* larvae are also a potential source of antimicrobial peptides (AMPs), with antibacterial activity against both Gram-positive and Gram-negative bacteria (46, 47). As probiotics, their physiological functions include enhancing digestion, regulating the balance of intestinal flora, improving immunity, and inhibiting the growth of harmful bacteria (48, 49). The alteration in intestinal bacteria populations and associated metabolites can affect the immune status of the host. The correlation analysis demonstrated that the changes in inflammatory cytokines, tight junction protein genes, and the SIgA concentration are associated with metabolites and specific bacteria, for example larvae as a potential dietary protein source altered the microbiota and modulated mucosal immune status in the colon of finishing pigs (7). Therefore, down-regulation of the expression levels of pro-inflammatory cytokines and up-regulation of the expression levels of anti-inflammatory cytokines and tight junction proteins confer the best form of resistance to ETEC K88.

**Figure 6:**
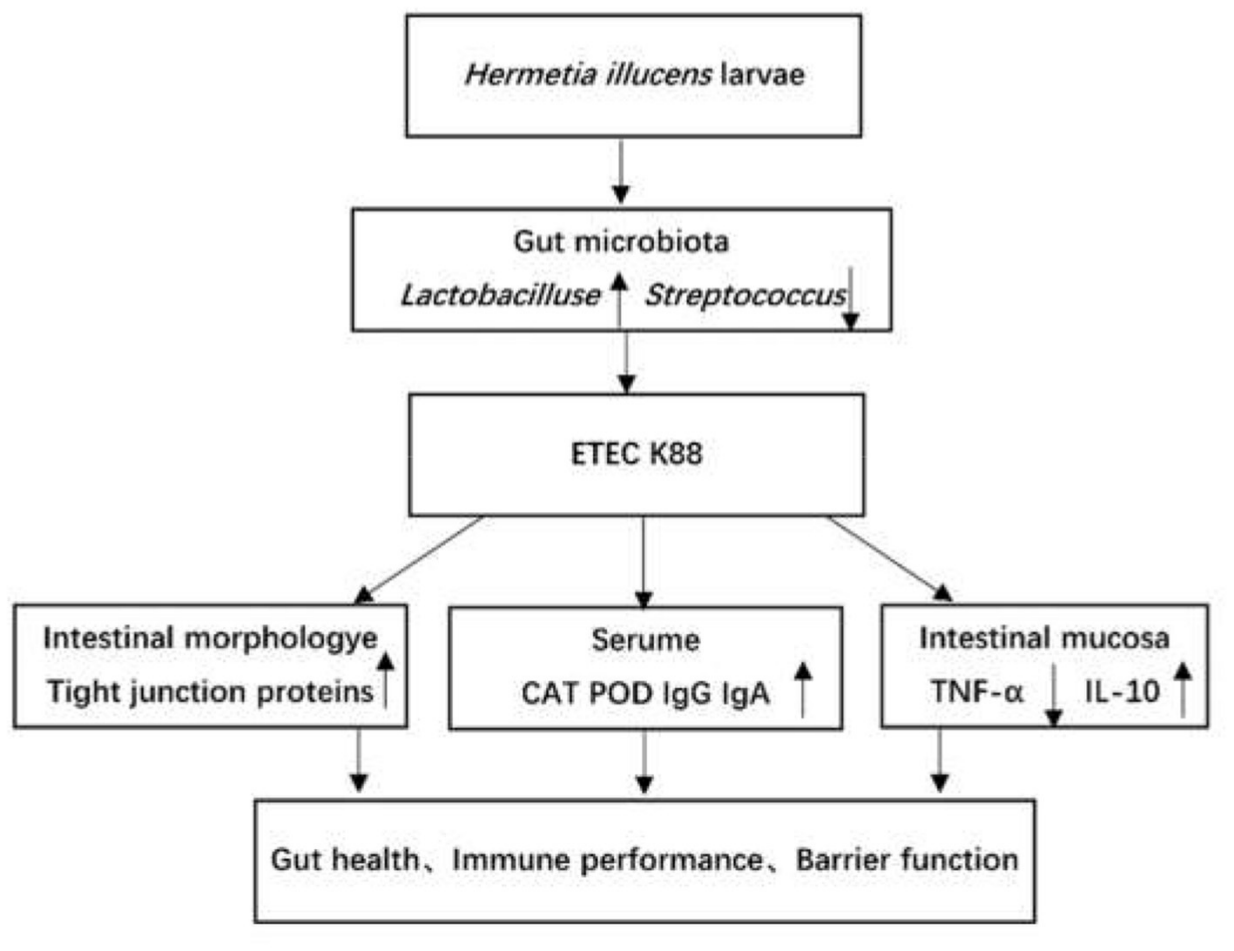
The overall picture shows the main results obtained in the current work. The up arrows (↑) indicate increasing effects, and the down arrow (↓) indicates decreasing effects.

Figure 6 summarizes the the main results obtained from this study. Nevertheless, the mechanism of *H. illucens* larvae powder on the altered intestinal flora and the influence on the intestinal barrier still needs further investigation.

## Conclusion

This study demonstrates that dietary supplementation of 4 and 8% *H. illucens* larvae did not affect the growth performance of weaned piglets. Notably, it can change the distribution of gut microbiota, increase the relative abundance of *lactobacillus*, and improve intestinal health. Adding 4% *H. illucens* larval to the piglets diet can increase the expression of tight junction proteins *Claudin-3* and *Occludin* and *IL-10* in the intestinal mucosa and maintain the ileum morphology's integrity, and portray a better immune status, improve intestinal barrier function. These studies provide a novel perspective for insect meals as a sustainable protein source for pig feed; the optimal dosage is 4%.

## Data Availability Statement

The datasets presented in this study can be found in online repositories. The names of the repository/repositories and accession number(s) can be found below: NCBI; PRJNA750611.

## Ethics Statement

The animal study was reviewed and approved by Animal Ethics Committee of Zhongkai University of Agricultural and Engineering. Written informed consent was obtained from the owners for the participation of their animals in this study.

## Author Contributions

WWa, YL, and YH designed research. XJ, WWu, and MZ conducted the pig trial. WWa, XJ, ML, BY, MZ, and HX prepared the diets. XJ, BY, ML, MZ, GX, and ZW performed research. WWa, XJ, ML, and XZ analyzed data. XJ, WWa, and YL wrote the manuscript. All authors read and approved the final manuscript.

## Funding

This work was supported by the National Natural Science Foundation of China (Nos. 31872442 and 31702150) and Innovative Team Projects of Ordinary Colleges and Universities in Guangdong Province (2020KCXTD019).

## Conflict of Interest

The authors declare that the research was conducted in the absence of any commercial or financial relationships that could be construed as a potential conflict of interest.

## Publisher's Note

All claims expressed in this article are solely those of the authors and do not necessarily represent those of their affiliated organizations, or those of the publisher, the editors and the reviewers. Any product that may be evaluated in this article, or claim that may be made by its manufacturer, is not guaranteed or endorsed by the publisher.

## Abbreviations

HI: Hermetia illucens
BW: Body weight
Cd: Crypt depth
CP: Crude protein
DE: Digestible energy
DM: Dry matter
EE: Ether extract
ADG: Average daily gain
F/G: Feed/Gain
ADFI: Average daily feed intake
Vh: Villus height
Cd: Crypt depth
Vh/Cd: Villus height to crypt depth ratio
PcoA: Principal coordinate analysis
SCFAs: Short-chain fatty acids.
