# Supplementary material for: Dietary Hermetia illucens Larvae Replacement Alleviates Diarrhea and Improves Intestinal Barrier Function in Weaned Piglets Challenged With Enterotoxigenic Escherichia coli K88
Source: Front Vet Sci. 2021 Nov 25;8:746224. doi: 10.3389/fvets.2021.746224 (PMC8655791; doi:10.3389/fvets.2021.746224)
Supplement: Supplementary file 1 [file Image_1.pdf]

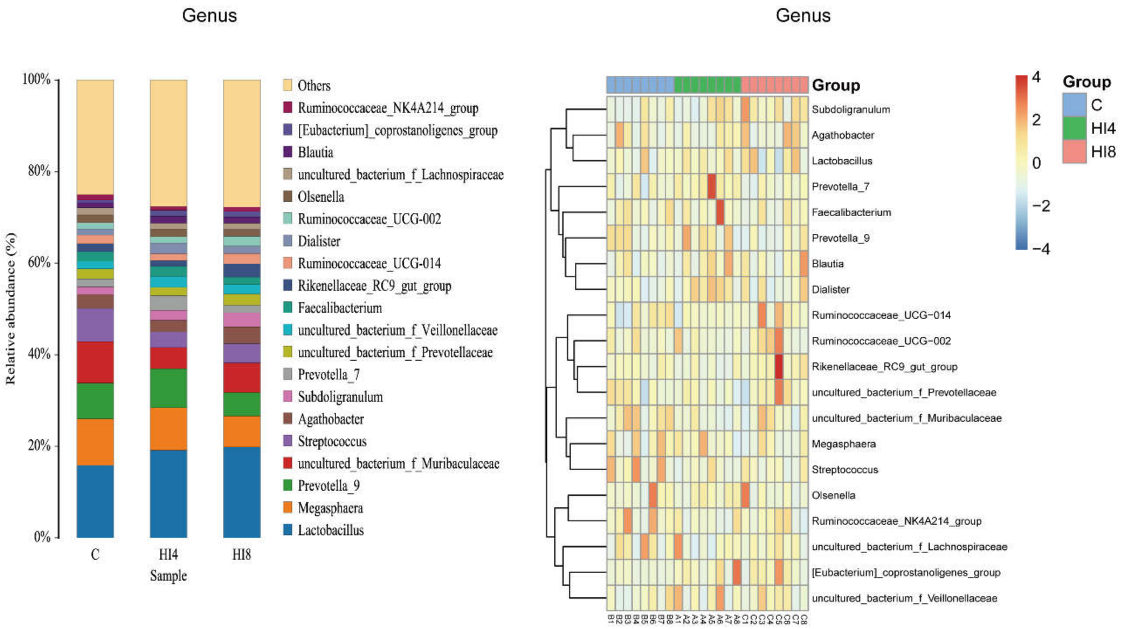

Figure S1. genus-level relative abundance of 16S rRNA sequences from the fecal of pigs and the heat map portrayed the 20 species of bacteria most dominant at the genus level in the feces. (n=8)
